# Mild Functional Differences of Dynamin 2 Mutations Associated to Centronuclear Myopathy and Charcot-Marie-Tooth Peripheral Neuropathy

**DOI:** 10.1371/journal.pone.0027498

**Published:** 2011-11-11

**Authors:** Olga S. Koutsopoulos, Catherine Koch, Valerie Tosch, Johann Böhm, Kathryn N. North, Jocelyn Laporte

**Affiliations:** 1 Department of Translational Medecine, IGBMC (Institut de Génétique et de Biologie Moléculaire et Cellulaire), Illkirch, France; 2 Inserm, U964, Illkirch, France; 3 CNRS, UMR7104, Illkirch, France; 4 Université de Strasbourg, Illkirch, France; 5 Institute for Neuroscience and Muscle Research, The Children's Hospital at Westmead, Sydney, Australia; 6 Discipline of Paediatrics and Child Health, Faculty of Medicine, University of Sydney, Australia; University Hospital Vall d'Hebron, Spain

## Abstract

The large GTPase dynamin 2 is a key player in membrane and cytoskeletal dynamics mutated in centronuclear myopathy (CNM) and Charcot-Marie Tooth (CMT) neuropathy, two discrete dominant neuromuscular disorders affecting skeletal muscle and peripheral nerves respectively. The molecular basis for the tissue-specific phenotypes observed and the physiopathological mechanisms linked to dynamin 2 mutations are not well established. In this study, we have analyzed the impact of CNM and CMT implicated dynamin 2 mutants using ectopic expression of four CNM and two CMT mutations, and patient fibroblasts harboring two dynamin 2 CNM mutations in established cellular processes of dynamin 2 action. Wild type and CMT mutants were seen in association with microtubules whereas CNM mutants lacked microtubules association and did not disrupt interphase microtubules dynamics. Most dynamin 2 mutants partially decreased clathrin-mediated endocytosis when ectopically expressed in cultured cells; however, experiments in patient fibroblasts suggested that endocytosis is overall not defective. Furthermore, CNM mutants were seen in association with enlarged clathrin stained structures whereas the CMT mutant constructs were associated with clathrin structures that appeared clustered, similar to the structures observed in *Dnm1* and *Dnm2* double knock-out cells. Other roles of dynamin 2 including its interaction with BIN1 (amphiphysin 2), and its function in Golgi maintenance and centrosome cohesion were not significantly altered. Taken together, these mild functional defects are suggestive of differences between CMT and CNM disease-causing dynamin 2 mutants and suggest that a slight impairment in clathrin-mediated pathways may accumulate over time to foster the respective human diseases.

## Introduction

Dynamins are large GTPases implicated in a variety of cellular processes including membrane and cytoskeletal dynamics as well as mitotic events. Classical dynamins were originally identified as microtubule interacting proteins [1] although their direct role in microtubule dynamics remains controversial [2], [3]. They are composed of a GTPase domain, a middle domain, a GTPase Effector Domain (GED), a Pleckstrin Homology domain (PH), and a Proline/arginine Rich Domain (PRD) that binds a plethora of proteins containing SH3 (Src homology 3) domains [4], [5]. Although the three classical dynamins (dynamins 1, 2 and 3) share high sequence identity they have different expression profiles and may have discrete roles. Dynamin 2 (DNM2) is ubiquitously expressed whereas dynamin 1 is predominantly expressed in brain and dynamin 3 has been detected in brain, testis and lung [6], [7], [8], [9], [10]. In humans, although we lack a complete expression profile for dynamin 2, it is expressed at least in skeletal muscle and peripheral nerve [11].

To date, only dynamin 2 has been implicated in disease out of the classical dynamins. *DNM2* mutations have been linked to two autosomal dominant diseases: centronuclear myopathy (CNM) and the axonal or intermediate form of dominant Charcot-Marie-Tooth (CMT) disease [12], [13], [14]. Most missense mutations linked to CMT are located within the PH domain while CNM with either adult or early onset has been linked to mutations in the middle and the PH domains, respectively [11], [15], [16], [17], [18]. The middle domain of dynamin 2 has been ascribed a role in centrosome cohesion [19] and seems to be important for dynamin oligomerization [20]. It has also been proposed to contribute to conformational changes induced by stimulation of GTP hydrolysis [21]. The PH domain is involved in phosphoinositide binding with a higher affinity for PtdIns(4,5)*P*_2_ [22]. Although several CNM and CMT-causing mutations have been identified in close proximity along the sequence of the *DNM2* gene [11] how they lead to distinct pathologies affecting discrete tissues remains an intriguing question. A possible concomitant expression of muscle weakness and nerve involvement was suggested in some patients although, in most cases, *DNM2* mutations have clear non-overlapping clinical outputs [23], [24]. Recently, it was shown that CNM mutations found within the PH domain increase the stability of the assembled form of dynamin but do not affect their lipid binding properties [25]. In contrast, tested CMT mutants displayed decreased oligomer stability [25]. Furthermore, middle domain CNM mutants were demonstrated to form more stable higher order polymers and displayed increased basal GTPase activity [26]. Therefore, it seems that CNM and CMT mutations affect dynamin 2′s biochemical properties in discrete ways. However, their effect on known cellular functions of dynamin 2 has not been addressed.

In this study, we set out to investigate the impact and differences between CNM and CMT mutations using ectopic expression and patient fibroblasts in established cellular processes of dynamin 2 action. Our observations suggest that CMT mutants maintain an interaction with MTs whilst CNM mutants lose this property. In addition, CNM mutant ectopic proteins are seen in association with enlarged clathrin-positive punctae. Although, an effect on clathrin-mediated endocytosis is apparent for all tested mutations after ectopic expression, albeit to different extents, experiments in patient fibroblasts suggest that this pathway is not largely affected in the related diseases.

## Results

### Differential impact of mutations on the subcellular localization of dynamin 2

**Figure 1:**
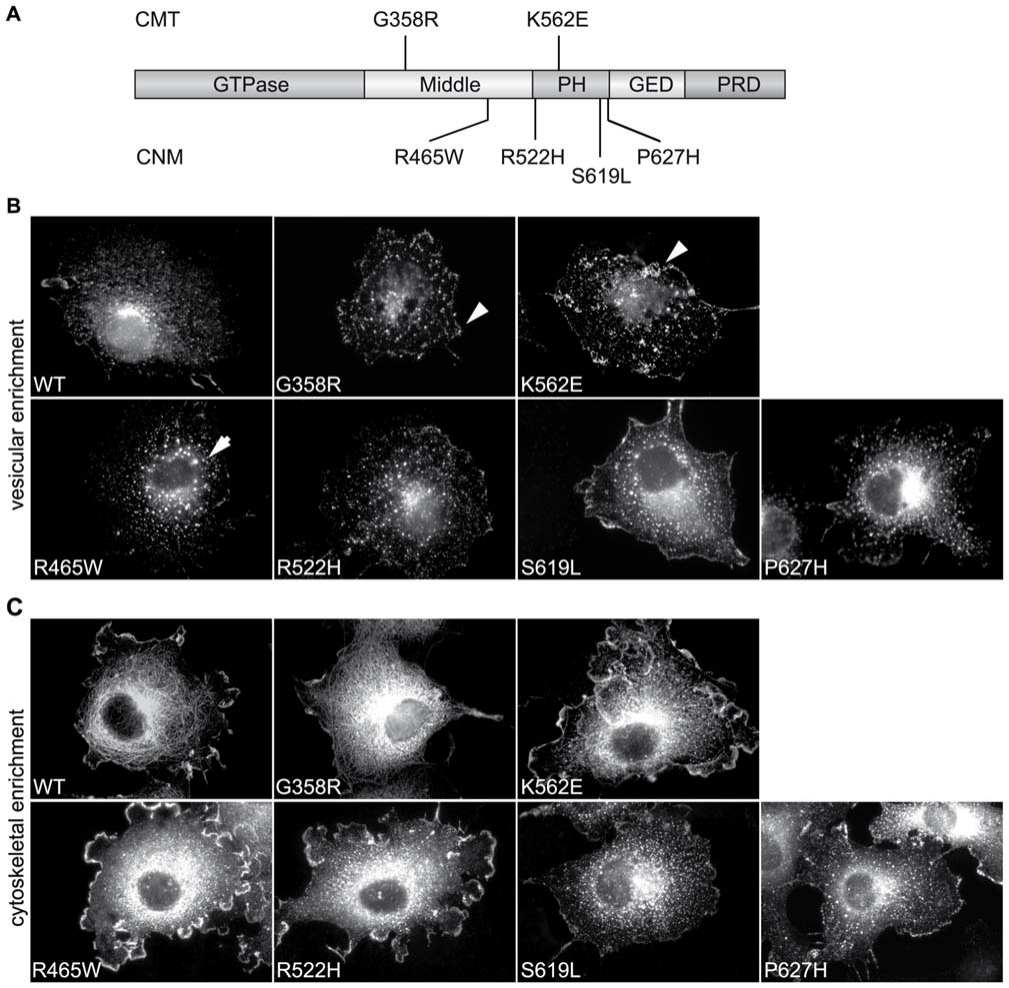
Localization profile of dynamin 2 CNM and CMT mutants. (**A**) Schematic representation of the dynamin 2 protein with protein domains depicted; GTPase, middle domain, PH: pleckstrin homology domain, GED: GTPase Effector Domain, PRD: proline-rich domain. Position of dynamin 2 mutations implicated in Charcot-Marie Tooth disease (CMT, above) and centronuclear myopathy (CNM, below) employed in this study are indicated. **(B)** Enrichment of dynamin 2 associated with vesicular structures. COS-1 cells transiently transfected with indicated dynamin 2 mutants were treated with 0.5% (v/v) Triton X-100, 2% (v/v) paraformaldehyde for 2 min at 37°C followed by fixation in paraformaldehyde and processing with anti-MYC specific antibodies. The subcellular localization profile for the wild type (WT) and indicated mutants is depicted. Clustered structures (arrowheads) and enlarged puncta (arrows) are indicated. (**C**) Enrichment of dynamin 2 along cytoskeletal elements. COS-1 cells transiently transfected with indicated dynamin 2 constructs were treated with Brinkley buffer containing 1% (v/v) Triton X-100 for 10 min at 37°C, to preserve cytoskeletal structures, followed by fixation in paraformaldehyde, and processed with anti-dynamin 2 specific antibodies. Wild type (WT) and CMT mutants display a filamentous profile not seen for CNM mutants.

In order to address the cellular impact of dynamin 2 mutations implicated in centronuclear myopathy and Charcot-Marie Tooth disease, we engineered constructs harboring patient mutations located within the middle and PH domain of dynamin 2 (Fig. 1A). As patients with dynamin 2-related diseases are heterozygous for the mutations, we chose to express these constructs in cells containing endogenous levels of wild type dynamin 2. COS-1 cells were transiently transfected with these constructs and were subjected to distinct treatments to compare the localization profile of both wild type (WT) and mutant dynamin 2 constructs. To remove cytosolic dynamin 2, the cells were treated with 0.5% (v/v) Triton X-100, 2% (v/v) paraformaldehyde ([27]; vesicular enrichment fixation). As can be seen in Fig. 1B, wild-type dynamin 2 localised to puncta throughout the cell and at the perinuclear region, potentially at the endosomal compartment and the Golgi network [6], [28], [29]. The staining profile for mutated dynamin 2 constructs was comparable, although the dynamin 2 vesicular structures appeared somewhat enlarged in cells expressing CNM mutants.

**Figure 2:**
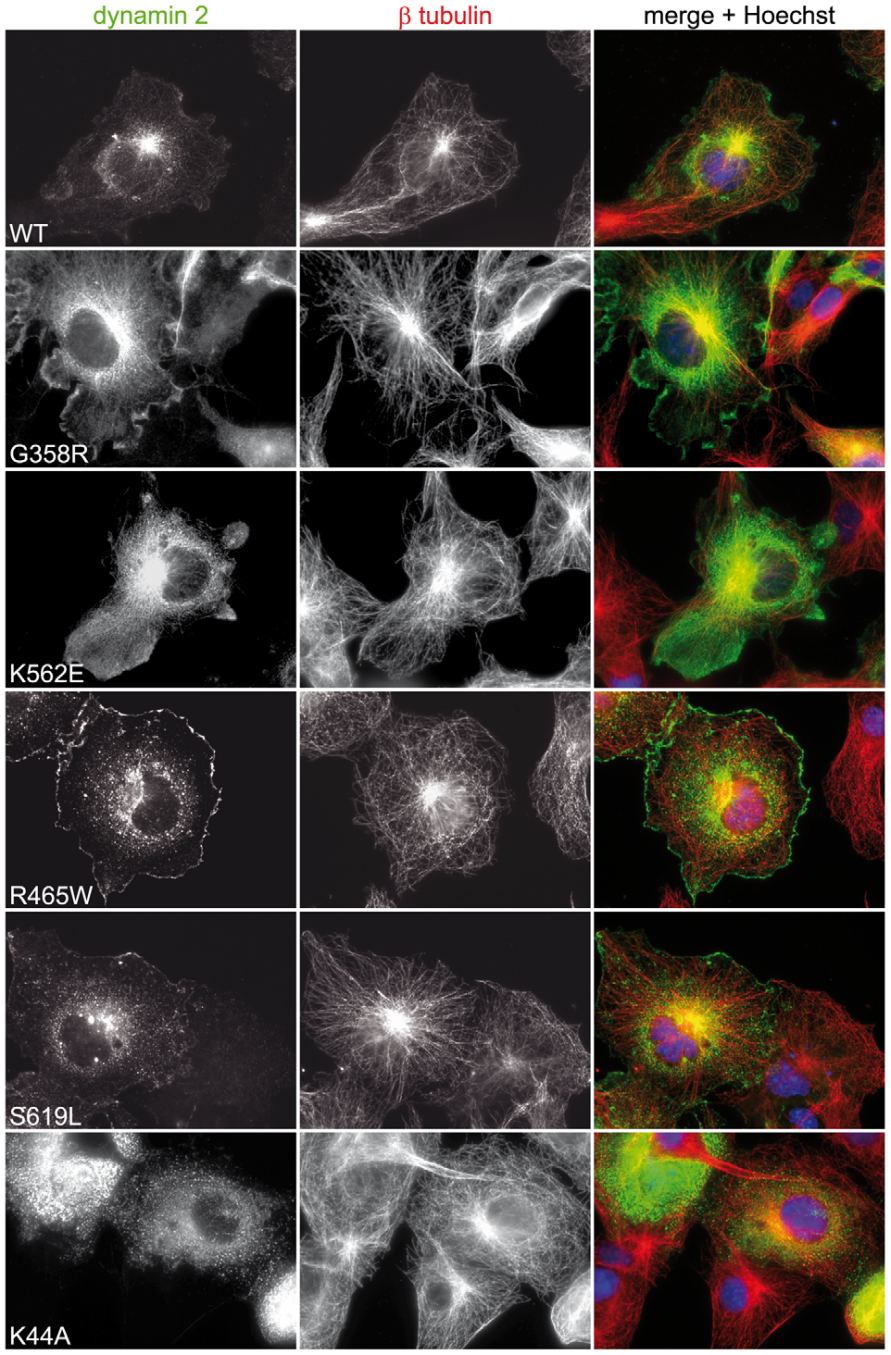
CNM mutations impact on dynamin 2′s localization to microtubules. COS-1 cells transiently transfected with the indicated constructs were treated with Brinkley buffer and 1% (v/v) Triton X-100, followed by fixation in paraformaldehyde and staining with anti-dynamin 2 and anti-β tubulin specific antibodies. The CMT mutants (G358R, K562E) display MT association, unlike dynamin 2 CNM (R465W, S619L) and K44A mutants that are seen decorating punctate structures.

**Figure 3:**
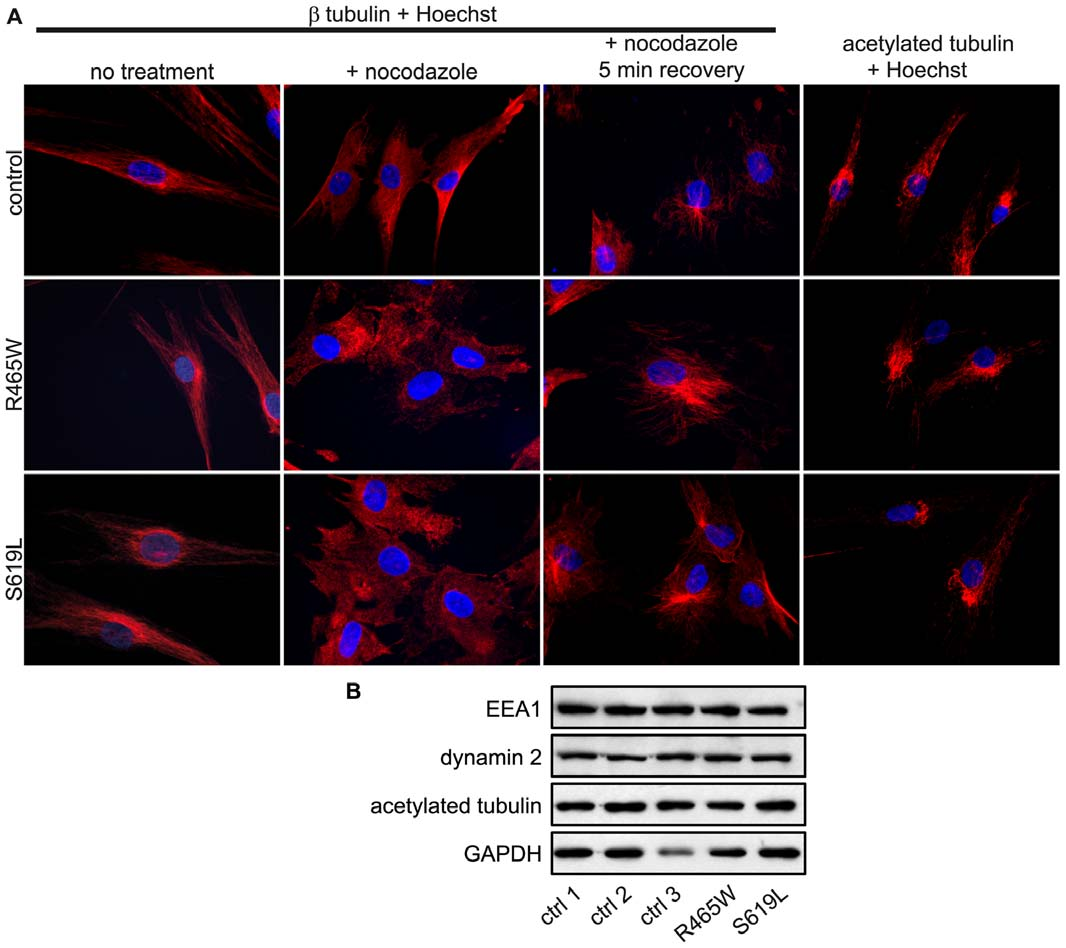
MT dynamics in CNM patient fibroblasts. **(A)** MT dynamics in fibroblasts from control and CNM patients harboring the R465W or S619L mutation. Fibroblasts were left untreated or were treated with 5 µM nocodazole for 2 h at 37°C. To allow for partial recovery of MTs, nocodazole was removed and cells were placed at 37°C for 5 min. To view MTs, the cells were stained with β tubulin specific antibodies. Stabilized MTs were visualized with acetylated tubulin-specific antibodies. (**B**) Levels of acetylated tubulin are comparable for control and patient fibroblast lines. Equal amounts of indicated fibroblast lysates were subjected to SDS-PAGE and were analyzed by western blot employing anti-acetylated tubulin antibodies and controls (anti-EEA1, anti-dynamin 2 and anti-GAPDH).

As ectopic dynamin 2 has been previously reported to associate with interphase microtubules (MTs), we treated cells expressing wild type or mutated dynamin 2 proteins with 1% (v/v) Triton X-100 in Brinkley buffer prior to fixation ([2], [3]; cytoskeletal enrichment fixation). This allows for preservation of the cytoskeleton and in particular of MTs, whilst it results in removal of cytosolic proteins. Under these conditions, wild type and CMT mutants dynamin 2 constructs formed a filamentous pattern, whereas the four dynamin 2 constructs harboring CNM mutations showed a punctate pattern throughout the cytoplasm (Fig. 1C), similar to the structures observed in Fig. 1B. As the filamentous pattern observed was reminiscent of MTs we co-stained for dynamin 2 and β tubulin under the same conditions and found that wild type and CMT mutants partially co-localized with MTs, while this co-localization was clearly decreased for dynamin 2 constructs harboring CNM mutations (Fig. 2 and Fig. S1). We were intrigued by this observation as a dynamin 2 construct with the CMT-associated 551Δ3 variation has been previously shown to display increased MT association [3]. Our observations suggested that, conversely, dynamin 2 CNM mutations may result in reduced dynamin 2 association with interphase MTs. The punctate pattern observed in the case of CNM mutants was examined further however we could not identify their nature. They did not co-label with EEA1, clathrin light chain, or proteasomal markers (data not shown). To explore the decreased MT association observed for dynamin 2 CNM mutants (see Fig. 2), we assessed MT morphology and dynamics in fibroblasts obtained from CNM patients harboring the R465W and S619L mutation, point mutations in the middle and PH domain of dynamin 2 respectively. These fibroblasts thus contain both wild-type and mutated alleles and express similar levels of dynamin 2 protein compared to control fibroblast lines as shown by western blot analysis (Fig. 3B). The MTs appeared normal for both patient lines as revealed by staining with β tubulin specific antibodies (Fig. 3A; left panels). Treatment of fibroblasts with nocodazole, an MT-depolymerizing drug, resulted in complete loss of MTs, with MT nucleation and elongation occurring similarly following drug removal for control and patient cells (Fig. 3A). Dynamin 2 was reported to affect the dynamic nature of MTs, as targeted disruption of *Dnm2* and *Dnm1* in mouse embryonic cells lead to an increased level of acetylated tubulin, a sub-population of stabilized α tubulin [30]. However, we observed no differences between control and patient fibroblasts by immunofluorescence (Fig. 3A; far right panels). No differences in the levels of acetylated tubulin were observed when these patient fibroblasts were compared to three control fibroblast lines by western blot analysis employing anti-acetylated tubulin antibodies and several control antibodies (Fig. 3B). Taken together, these observations indicate a differential impact of CMT and CNM mutations on the subcellular localization of dynamin 2, while they do not readily affect interphase MT dynamics.

### Impact of dynamin 2 mutations on clathrin-mediated endocytosis

**Figure 4:**
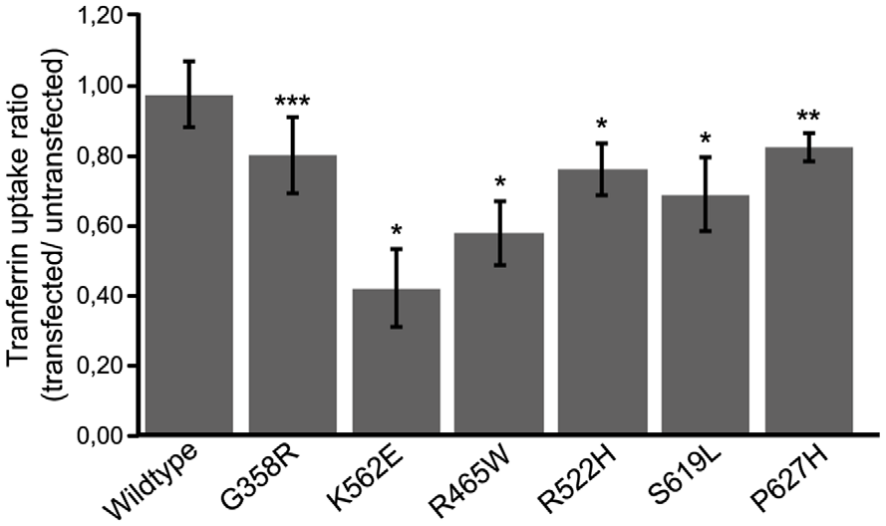
Effect of dynamin mutants' overexpression on transferrin uptake. COS-1 cells expressing ectopic wild type and mutant dynamin 2 constructs were incubated with fluorescently labeled transferrin for 15 min and were subsequently fixed and processed with anti-MYC antibodies for microscopic observation. Quantification of transferrin uptake employing the Metamorph software allowed for calculation of the ratio of the intensity of transfected cells/untransfected cells representing the correlation of three independent experiments. The student's *t* test was used for statistical analysis of three independent experiments, * *P*<0.01, ** *P*<0.05, *** *P*>0.05.

Dynamin has an established role in clathrin-mediated endocytosis (CME) [31]. To investigate the effect of disease-related dynamin 2 mutants on this process, we monitored CME in cells transfected with the various mutants following incubation with fluorescently labeled transferrin. As shown in Fig. 4 and Fig. S2, we observed a significant decrease in transferrin uptake for all the mutants tested except for the G358R mutation that is related to a CMT phenotype. This decrease was more pronounced for the K562E CMT mutation (decrease by approximately 50% compared to wild type dynamin 2).

**Figure 5:**
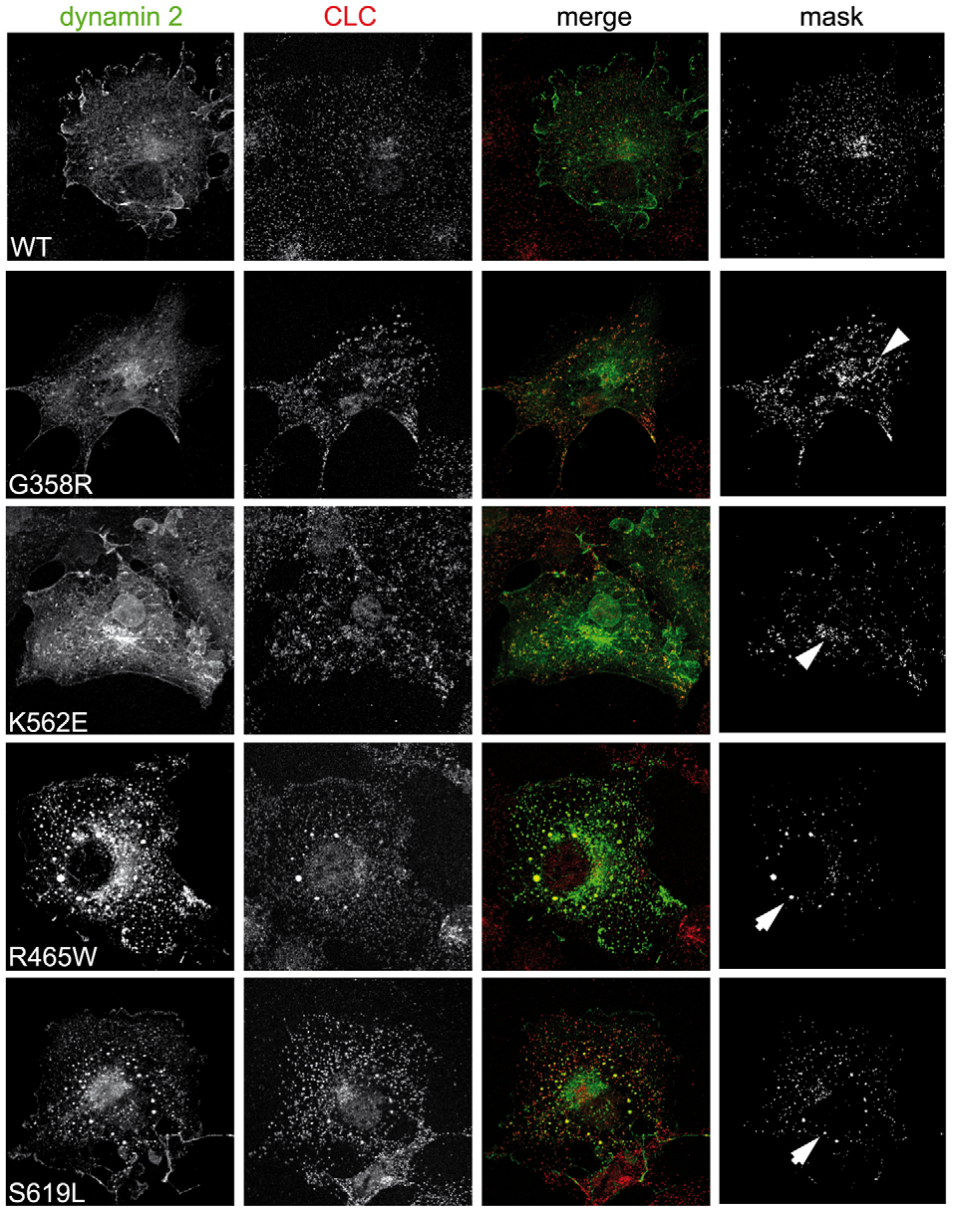
Comparison of dynamin 2 mutants' localization to clathrin-positive structures following a vesicular enrichment fixation. COS-1 cells transiently transfected with indicated dynamin 2 mutants were treated with 0.5% (v/v) Triton X-100, 2% (v/v) paraformaldehyde for 2 min at 37°C followed by fixation in paraformaldehyde and processing with anti-MYC specific antibodies and anti-clathrin light chain (CLC) antibodies. Samples were analyzed by confocal microscopy. A mask for the colocalization that was created employing the Fiji software is shown (right panels). Note the presence of aggregated vesicles in dynamin 2 G358R and K562E-expressing cells (arrowheads) and the presence of enlarged clathrin-positive punctae (arrows) in the case of R465W and S619L mutant expressing cells.

We next tested whether ectopic expression of CNM and CMT dynamin 2 mutant constructs can reveal subtle effects on the morphology of clathrin positive structures. Co-staining of cells overexpressing the various mutants with anti-dynamin 2 and clathrin light chain (CLC) antibodies revealed differences for the two classes of mutants. CMT mutants were seen in association with clustered punctae whereas CNM mutants were seen in association with enlarged clathrin positive structures (Fig. 5 and Fig. S3). The clustered structures observed in the case of the K562E expressing cells were reminiscent to the clustering of clathrin coated vesicles observed in conditional mouse embryonic fibroblasts lacking *Dnm1* and *Dnm2* expression (Fig. S3) and as previously reported [30]. On the other hand, the appearance of enlarged structures co-labeled for both dynamin 2 and clathrin in cells overexpressing CNM mutants would be consistent with increased fusion events or a decrease in fission events.

**Figure 6:**
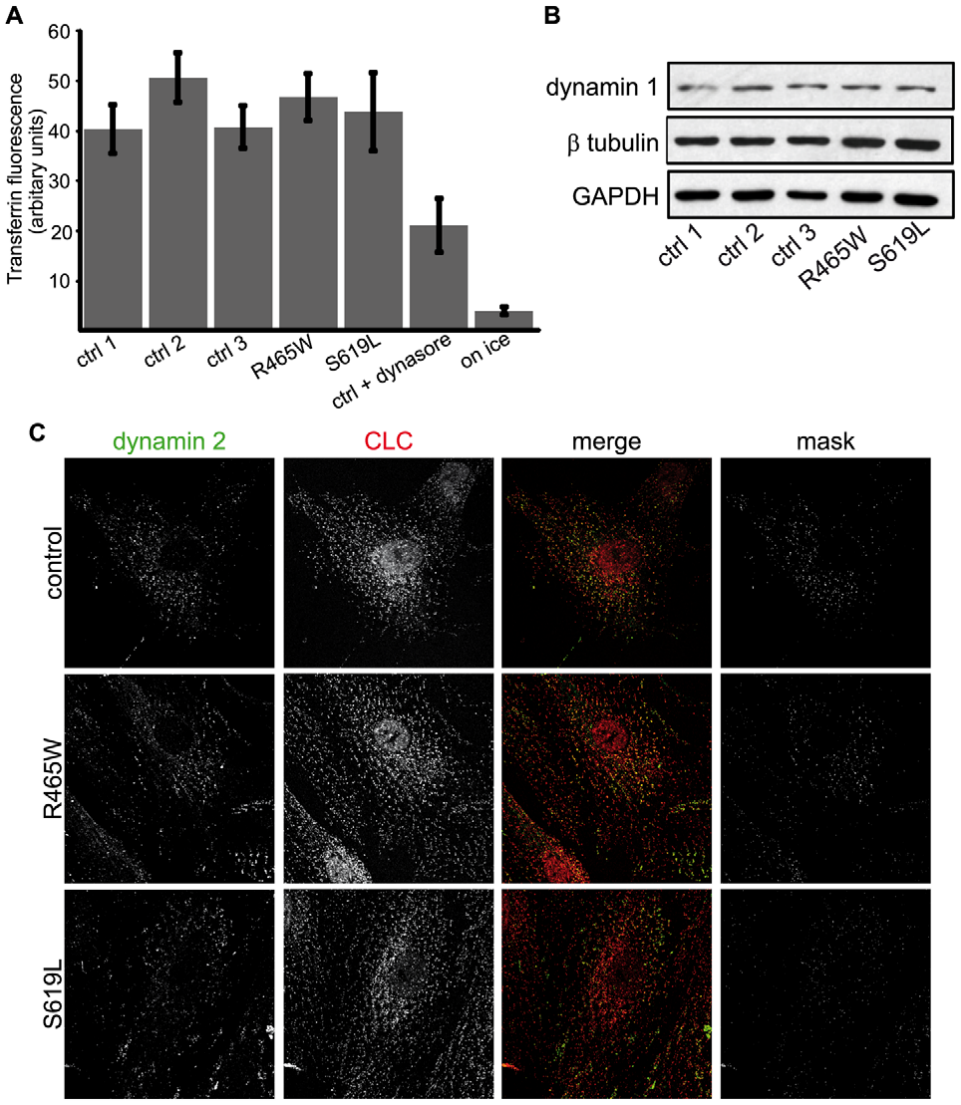
CNM mutations do not impact on transferrin uptake in patient fibroblasts harboring the R465W or S619L mutation. **(A)** Quantification of transferrin uptake. Control and patient fibroblasts were serum-starved and incubated with fluorescently labeled human transferrin for 15 min at 37°C. Levels of internalized transferrin were analysed by FACS following removal of non-internalized transferrin. Error bars represent standard deviation for three independent expreriments. The student's *t* test was used for statistical analysis of three independent experiments, *P*<0.05 were considered significant. **(B)** Levels of dynamin 1 in control and patient fibroblasts. Equal amounts of control and patient (R465W and S619L) fibroblasts were analyzed by western blot employing anti-dynamin 1 and indicated control antibodies. **(C)** Endogenous dynamin 2 co-localizes with clathrin light chain (CLC). Control and patient fibroblasts (R465W and S691L) were fixed in 0.5% (v/v) Triton X-100, 2% (v/v) paraformaldehyde. Right panels depict masks corresponding to the co-localization of anti-dynamin 2 and anti-CLC that were generated employing the Fiji program.

To investigate whether these observations reflect an impact on dynamin's role in CME, we investigated this process in patient fibroblasts with the R465W or S619L CNM mutations by measuring the levels of internalized fluorescently labeled transferrin following serum deprivation. While dynasore or incubation at +4°C substantially decreased transferrin uptake; untreated control and patient fibroblasts displayed similar levels of transferrin internalization (Fig. 6A). As endogenous dynamin 2 protein levels were comparable for patient and control fibroblasts employed in our study (see Fig. 3B), we envisaged that a potential upregulation of dynamin 1 could make up for and hence mask an impact on endocytosis in the patient fibroblasts assessed. Dynamin 1 has been shown to be upregulated in fibroblasts lacking dynamin 2 [30] and can compensate to some extent for dynamin 2′s role in endocytosis. The protein levels of dynamin 1, albeit low in fibroblasts, were similar for all the cell lines analyzed (Fig. 6B) suggesting that dynamin 1 is not upregulated in the R465W and S619L fibroblast lines and thus could not differentially influence CME. In addition, dynamin 2 was found in association with clathrin coated vesicles similarly in control and patient fibroblasts, following fixation with 0.5% (v/v) Triton X-100, 2% (v/v) paraformaldehyde, suggesting that dynamin's role in endocytosis is not altered in patient cells (Fig. 6C).

### Dynamin 2 recruitment to BIN1-induced membrane tubules

**Figure 7:**
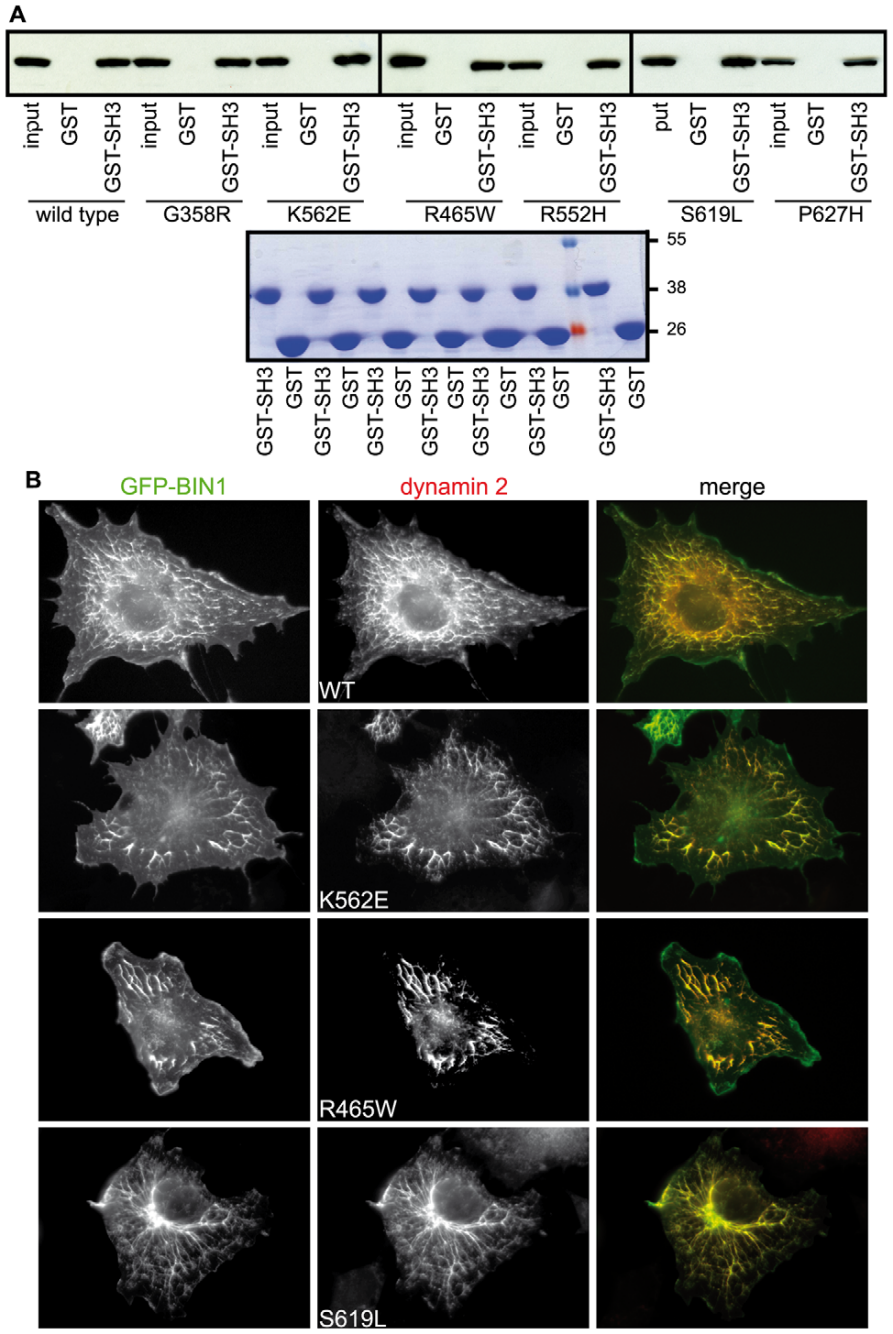
CNM and CMT dynamin 2 mutations do not impact on BIN1 (amphiphysin 2) interaction. **(A)** CNM and CMT mutants interact with the BIN1 SH3 domain. Lysates from COS-1 cells transiently transfected with constructs encoding indicated dynamin 2 mutants were subjected to pull-down assays employing recombinant GST-SH3 (BIN1) or GST alone coupled to glutathione beads. The samples were analyzed by SDS-PAGE and were subjected to western blot analysis employing anti-MYC specific antibodies. The input fraction represents 5% of total material employed in the pull-downs. Below, Coomassie Blue staining showing recombinant proteins coupled to beads employed for pull-downs. (**B**) Recruitment of dynamin 2 constructs to BIN1-induced membrane tubules. COS-1 cells were transiently transfected with vectors encoding indicated dynamin 2 constructs and the GFP-BIN1 chimera. Cells were fixed in paraformaldehyde followed by staining with anti-MYC specific antibodies. Both CNM and CMT dynamin 2 mutants can be seen in association with BIN1 tubules.

BIN1 (amphiphysin 2), a dynamin 2-interacting partner with an established role in clatrhin-mediated endocytosis, has been found mutated in recessive forms of CNM [32]. In particular, a disease-causing BIN1 mutation was reported to strongly decrease BIN1 binding to dynamin 2, suggesting that a disruption of the BIN1-dynamin 2 complex may play a role in the CNM pathology [32]. To test if CNM and/or CMT-linked dynamin 2 mutations could interfere with dynamin 2-BIN1 interactions, we performed pull-down assays employing lysates from COS-1 cells transfected with CNM or CMT dynamin 2 mutant constructs that were combined to recombinant GST-SH3 (BIN1) or GST proteins coupled to glutathione beads. All dynamin 2 mutants were pulled-down efficiently with the SH3 domain of BIN1, suggesting that the mutations do not impact on this interaction at least under the conditions implemented (Fig. 7A). These results are consistent with the fact that the main BIN1-binding site on dynamin 2 is the Proline-rich domain [33], [34]. As BIN1 induces the formation of tubules when over-expressed in COS-1 cells [32] we next assessed whether mutations in dynamin 2 disrupt its recruitment to BIN1-induced membrane tubules. Wild type dynamin 2 localized to BIN1-induced tubules (Fig. 7B, top panels). All CNM and CMT dynamin 2 mutants tested were also seen in association with tubules decorated with BIN1 confirming our pull-down findings. BIN1-induced tubules persisted following recruitment of ectopic dynamin 2 under these conditions (Fig. 7B and Fig. S4).

### Dynamin 2 mutants do not affect centrosome cohesion or Golgi maintenance

**Figure 8:**
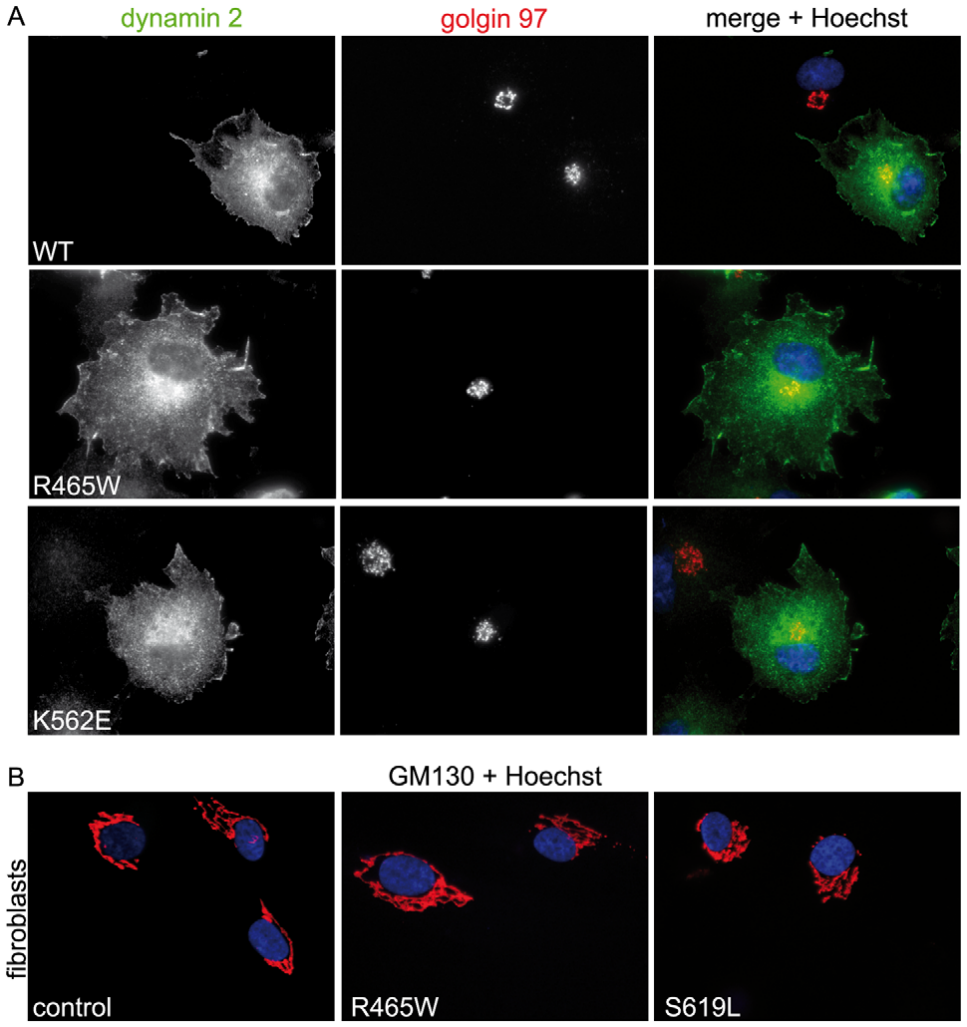
Effect of dynamin 2 mutations on Golgi network morphology. **(A)** Overexpression of CNM and CMT dynamin 2 mutants does not impact on Golgi morphology. COS-1 cells expressing ectopic dynamin 2 constructs were fixed in paraformaldehyde and were stained with anti-dynamin 2 and anti-golgin 97 antibodies. **(B)** Golgi network morphology is comparable between control and patient fibroblasts harboring CNM mutations. Fibroblasts were fixed in paraformaldehyde and were stained with anti-GM130 antibodies and Hoechst.

**Figure 9:**
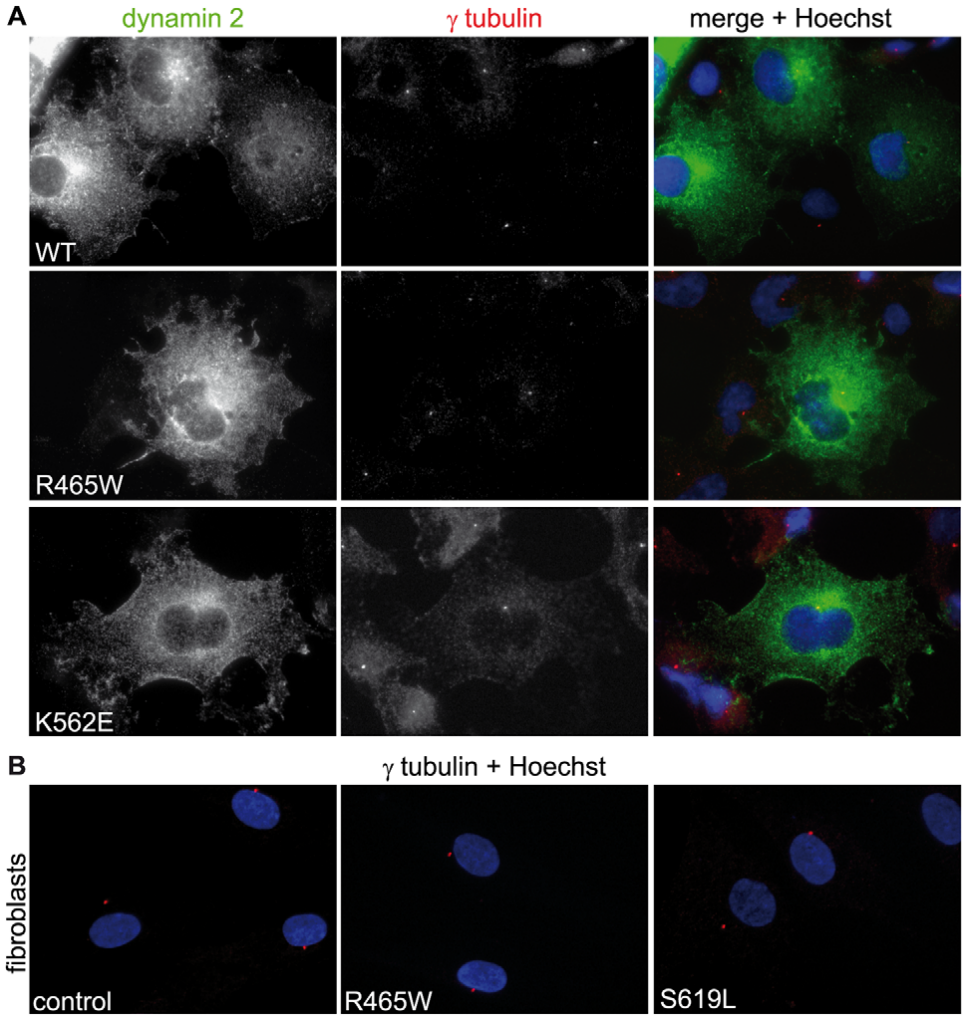
Effect of dynamin 2 mutations on centrosome cohesion. **(A)** Overexpression of CNM and CMT dynamin 2 mutants does not impact on centrosome cohesion. COS-1 cells expressing ectopic dynamin 2 constructs were fixed in methanol:acetone and were stained with anti-dynamin 2 and anti-γ tubulin antibodies. **(B)** Centrosome position is comparable between control and patient fibroblasts harboring CNM mutations. Control and patient fibroblasts harboring CNM mutations were fixed in methanol:acetone and were stained with anti-γ tubulin antibodies and Hoechst.

To investigate if other known functions of dynamin 2 are affected by the CMT and CNM mutations, we monitored its role in Golgi network maintenance [3], [28], [35], [36] and centrosome cohesion [13], [19]. Overexpression of the mutants in COS-1 did not affect Golgi morphology as revealed by staining with anti-golgin 97 antibodies (Fig. 8A and data not shown). No effect on Golgi morphology was evident in CNM fibroblasts (R465W and S619L) either (Fig. 8B). Overexpression of wild type or mutated dynamin 2 constructs in COS-1 cells did not affect centrosome positioning (Fig. 9A and data not shown). In addition, we did not observe any differences in the organization of centrioles in the R465W or S619L patient fibroblasts when compared to control fibroblasts by staining for γ tubulin following methanol:acetone fixation (Fig. 9B), and as previously reported for the R465W mutation [13]. These observations suggest that the dynamin 2 mutations tested do not readily affect Golgi maintenance or centrosome positioning.

## Discussion

Although dynamin 2 mutations lead to two discrete pathologies affecting different tissues, the molecular basis of this specificity is not understood. It seems that, for example, dynamin 2 mutations in the middle and PH domains leading to CNM increase its GTPase activity through potentially the stabilization of higher order polymers [25], [26]. Conversely, the K562E CMT mutation displays decreased GTPase activity [25]. It is not unreasonable to think that changes in the properties and assembly-state of dynamin 2 observed in *in vitro* assays may be relevant to the effect of these mutations at the cellular level. In this study, we have analyzed the impact of both CNM and CMT-causing mutations on characterized cellular pathways of dynamin 2 action. We tested the impact of these mutant proteins in the presence of endogenous wild type dynamin 2, both in the ectopic expression studies and at the endogenous level in patient fibroblasts, to be closer to the physiological conditions observed in the associated pathologies as patients are heterozygous for the mutations. We did not observe any effect on Golgi maintenance and centrosome cohesion, two reported dynamin 2 functions [19], [37], suggesting that other functions are affected in the linked diseases.

Although we observed a clear loss of MT association in the case of CNM mutant proteins, we could not detect a direct impact on MT dynamics in patient cells. An increase in acetylated tubulin (a sub-population of stable MTs) has been previously reported in *Dnm2* knockout mouse embryonic fibroblasts [30], in HeLa cells expressing *DNM2* targeting siRNA [3], in flies expressing the *shibire* temperature sensitive mutant of dynamin [38], and in cells overexpressing the 551Δ3 CMT mutant [3]. However, consistent with our observations, Tanabe and Takei did not observe an effect on acetylated tubulin when they expressed the K562E CNM mutant [3]. Thus, defects in MT dynamics appear to be linked either to loss of the dynamin 2 protein or the deletion of the PRD domain [2], [30]. The 551Δ3 CMT mutant could still bind to MTs and exert a potential dominant negative effect thus resulting in increased MT stability however, we did not observe an effect on MT dynamics in the patient fibroblasts assessed. The presence of a normal copy of dynamin 2 in the patient cells is probably sufficient to sustain normal MT dynamics. However, we cannot exclude that an effect on MT stabilization and dynamics could become apparent in muscle cells during myotube formation as MT dynamics have been implicated in muscle cell differentiation [39], [40]. Alternatively, the dynamin 2 CNM mutations tested here may not impact on MT dynamics but on other dynamin 2 related pathways, as suggested by our results.

An impact on CME has been reported previously for both CNM and CMT dynamin 2 mutations [3], [11], [14]. We confirmed a partial inhibition of CME in COS-1 cells overexpressing CMT and CNM mutants by approximately 30% to 50% (see Fig. 4), except for the G358R CMT mutant for which the decrease in transferrin uptake was not significant. However, an effect on CME was not evident in patient fibroblasts harboring the R465W or the S619L CNM-related mutations. Interestingly, Bitoun and colleagues noted a decrease in CME in a cell line with the R465W CNM mutation after 5 minutes of uptake [11]; taken together with our results it suggests that dynamin 2 mutations delay but do not block endocytosis, at least in the case of this mutation. The apparent discrepancy in the transferrin uptake rate between the patient fibroblasts and the overexpression of the equivalent mutant constructs suggests that the relative abundance of the mutant protein compared to wild type dynamin 2 protein potentially influences the ability of dynamin 2 to readily participate in CME. In the case of the overexpression in COS-1 cells we probably have an excess of ectopically expressed dynamin 2 compared to endogenous protein suggesting that either a high level of mutant or an accumulation of the mutated protein over time in patients may cause partial CME defects. It can also reflect an accumulation of a minor cellular defect over time in the affected tissue. This is supported by the surprising lethality of the dynamin 2 (R465W) homozygous knock-in and the CME defect observed in homozygous knock-in MEFs that was absent from heterozygous knock-in MEFs [41]. It is noteworthy however, that the clathrin-positive structures observed following overexpression of the two different classes of mutant dynamin 2 proteins were distinct (see Fig. 5). CNM-related dynamin 2 mutants were seen in association with enlarged clathrin-positive structures. Taken together with our previous observations on the mis-localization of these mutants (see Fig. 2 and Table 1), it suggests that CNM mutations reduce dynamin 2′s ability to associate with MTs by either affecting their ability to bind to MTs or by decreasing their availability for binding to MTs due to increased association with clathrin-positive structures. Furthermore, the CMT-causing mutations lead to the formation of clustered structures reminiscent to the arrested pits observed in dynamin knockout cells [30]. Potentially this is linked to the decreased GTPase activity reported for the K562E mutant in the presence of liposomes [25], which may render the protein less efficient at exerting its function during clathrin coated vesicle maturation. In addition, the co-localization of the dynamin mutants to clathrin positive structures and the recruitment of all dynamin mutants to BIN1-induced tubules suggest that it is not a defect in dynamin's recruitment to clathrin coated pits *per se* but most likely a downstream event. Furthermore, while some amphiphysin 2 mutations leading to CNM disrupt the binding and recruitment of dynamin 2 to BIN1-induced membrane tubules [32], we show here that the dynamin 2 mutations tested do not have a similar impact. This is important for a better understanding of the pathophysiology of such diseases and suggests that dynamin 2 is downstream of amphiphysin 2 in this pathway.

**Table 1:** Comparison of the dynamin 2 disease-causing mutations tested.

Disease	CMT		CNM			
Mutations	G358R	K562E	R465W	R522H	S619L	P627H
Affected domain	Middle	PH	Middle	PH	PH	PH
Localization to MT's^1^	+	+	-	-	-	-
MT dynamics^2^			MT stability not affected		MT stability not affected	
CME and clathrin-positive structures^1^	normal and clustered	strongly decreased and clustered	decreased and enlarged	decreased and enlarged	decreased and enlarged	decreased and enlarged
CME in patient cells^2^			normal		normal	
Golgi maintenance^1,2^	normal	normal	normal	normal	normal	normal
Centrosome cohesion^1,2^	normal	normal	normal	normal	normal	normal

1 ectopic expression, ^2^in patient fibroblasts.

Given the relative mild phenotype associated with the so far identified dynamin 2 mutations leading to disease, compared to the embryonic lethality of the dynamin 2-null mouse between embryonic days 8 and 12 [30], it is not surprising that they seem to lead to a partial disruption of dynamin's function. These observations are supported by the recent study characterizing the first *Dnm2* knock-in mouse model [41]. Although the heterozygous R465W knock-in mouse is viable and recapitulates features of CNM, the homozygous knock-in is perinatal lethal [41]. This suggests that the wild type protein can rescue most of the effect of the mutated allele. Ectopic expression of this CNM mutation in wild type mice with an adeno-associated viral vector (AAV) supported a dominant effect of the mutation on muscle fiber structural maintenance. Importantly, only skeletal muscles were transduced in this model [42]. As muscles from the R465W KI mouse, where all tissues express the mutated protein, displayed very similar defects to the AAV model, it points to a muscle-specific impact of the CNM mutations. Animal models for the CMT disease linked to dynamin 2 mutations are not yet available and will be very valuable to decipher dynamin 2 pathways implicated in disease and their tissue-specific pathological mechanisms.

## Materials and Methods

### Cloning and constructs

The generation of the wild-type human dynamin 2 (isoform 1; Accession number NM_001005360) pENTR1A construct and the mammalian expression vector pTL1 clones have been reported elsewhere [32]. Human dynamin 2 CMT (G358R and K562E), CNM (R465W, S619L, R522H and P627H) and the artificial (K44A) mutant constructs were generated by primer directed PCR mutagenesis. Mammalian expression C-terminal myc-His tagged constructs were generated by recombination employing the Gateway® system (Invitrogen). The construction of pEGFP-C1-BIN1 and pGEX2T-SH3 (BIN1) has been previously described [32]. All constructs were verified by direct sequencing.

### Cell culture

Control and patient (R465W and S619L mutations) fibroblasts were grown in Dulbecco's modified medium (DMEM) supplemented with 10% (v/v) fetal calf serum (FCS) at 37°C in a humidified incubator, 5% CO_2_. Conditional *Dnm1* and *Dnm2* double knock-out fibroblasts, a kind gift from S. Ferguson and P. De Camilli, were grown in DMEM supplemented with 10% FCS in a humidified incubator supplemented with 5% CO_2_ at 37°C [30]. Deletion of *Dnm1* and *Dnm2* was induced by treatment with 4-hydroxytamoxifen (Sigma Aldrich) as specified by Ferguson *et al*. (2009). COS-1 cells were grown in DMEM supplemented with 5% (v/v) FCS under the same conditions and were transiently transfected with expression plasmids using LipofectAmine 2000 according to manufacturer's instructions (Invitrogen). Equal amounts of protein for control and patient fibroblasts were resuspended in Laemnli loading dye and analyzed by Tris-Glycine SDS-PAGE and western blot analysis. Antibodies employed included anti-GAPDH (Millipore), anti-acetylated tubulin (Sigma Aldrich), anti-dynamin 1 (Epitomics), anti-dynamin 2 (R2680; IGBMC [42]) and anti-β tubulin (clone 1TUB2A; IGBMC).

### Immunofluorescence assays

Cells were seeded directly onto sterilized coverslips. For γ tubulin staining, cells were fixed in cold methanol:acetone for 20 min at −20°C. For dynamin localization to MTs, cells were incubated in Brinkley buffer (80 mM PIPES pH 7.5, 1 mM MgCl_2_, 4% (w/v) polyethylene glycol, 1% (v/v) Triton X-100) for 10 min at 37°C followed by fixation in paraformaldehyde as described in [3]. For removal of cytosolic proteins, cells were fixed in 0.5% (v/v) Triton X-100, 2% (v/v) paraformaldehyde (EMS) for 2 min as specified in [27]. BIN1-induced tubules were preserved by fixing COS-1 cells in 2% paraformaldehyde (v/v) for 1 h at room temperature. For nocodazole treatment, fibroblasts were left untreated or were treated with 5 µM nocodazole (Sigma Aldrich) for 2 h at 37°C. For microtubules network recovery experiments, the drug was removed, cells were rinsed in pre-warmed cultured media and microtubules were allowed to recover for 5 min at 37°C. Antibodies employed in immunofluorescence assays included anti-golgin 97 (Invitrogen), anti-GM130 (BD Biosciences), anti-γ tubulin (Santa Cruz Biotech), anti-acetylated tubulin (Sigma Aldrich), anti-clathrin light chain (Sigma Aldrich), anti-EEA1 (Abcam), anti-β tubulin (clone 1TUB2A; IGBMC) and anti-dynamin 2 antibodies (R2641; IGBMC). These antibodies were revealed by employing anti-rabbit AlexaFluor 488 conjugated or anti-mouse AlexaFluor 594 conjugated secondary antibodies (Invitrogen). Nuclei were stained with Hoechst 33528 (Sigma Aldrich) and samples were mounted in FluorSave^TM^ reagent (Calbiochem).

### FACS analysis of transferrin uptake in fibroblasts

Serum-deprived fibroblasts were pulsed with AlexaFluor 633 conjugated transferrin (Invitrogen) for 15 min. Cells were washed twice for 3 min in 0.2 M acetic acid, 0.5 M NaCl followed by washing in 0.25 M Tris (pH 10) for 2 min and were subsequently fixed in 1% (v/v) formaldehyde. To block dynamin action, cells were treated with dynasore (Sigma Aldrich) as described by Macia *et al*. (2006) [43]. The samples were analyzed on the FACSCalibur (BD Biosciences) employing the Cell Quest Pro program (BD Biosciences). Subsequent analysis was performed employing the Flowjo software (Tree Star Inc., Oregon, USA). The student's *t* test was used for statistical analysis. P values of <0.01 were considered significant. To calculate the % of transferrin uptake, the fluorescence of the control with the highest uptake was noted as 100%. The % of uptake for all other cell lines was calculated as a ratio against the control cells.

### Transferrin uptake in transfected COS-1 cells

COS-1 cells transiently transfected with dynamin 2 wild type or mutant constructs were serum-starved for 1 h prior to treatment with AlexaFluor 488 conjugated transferrin for 15 min at 16 h post-transfection. Cells were fixed, permeabilized and processed with anti-MYC antibodies. Images were acquired using a DMRXA2 microscope (Leica Microsystems Gmbh). Quantification was performed employing the Metamorph software (Molecular Devices Inc., Sunnyvale, USA).

### Imaging and data processing

Imaging of mammalian cells was performed on a fluorescence microscope DM4000B (Leica Microsystems Gmbh) fitted with a colour CCD camera (Coolsnap cf colour, 180 Photometrics) or with a confocal laser scanning microscope (SP2; Leica Microsystems Gmbh) on an upright DMRXA2 microscope using the 63×1.4 oil immersion lens. Data processing was performed using ImageJ (Rasband, W.S., ImageJ, U.S. National Institutes of Health, Bethesda, Maryland, USA, http://rsb.info.nih.gov/ij/, 1997–2009) or Adobe Photoshop CS2 (Adobe Systems Incorporated). The mask was generated using the Fiji program (http://pacific.mpi-cbg.de/wiki/index.php/User:Schindelin).

### Pull-down assays

Recombinant GST and GST-SH3 (BIN1) were expressed in the Rosetta BL21 cell line. The recombinant proteins were purified employing glutathione coupled beads (GE Healthcare). COS-1 cells transiently transfected with WT or mutant human dynamin 2 constructs were solubilized in lysis buffer [20 mM HEPES-KOH (pH 7.4), 100 mM NaCl, 1 mM MgCl_2_, 0.5% (v/v) Triton X-100, 1 mM EGTA, 1 mM DTT, 5% (v/v) glycerol with a protease inhibitor cocktail (Roche)] for 1 h at 4°C. The soluble fraction that was recovered following centrifugation at 20,000 x *g* for 30 min at 4°C was combined to glutathione beads coupled to GST or GST-SH3 (BIN1) overnight. Unbound proteins were removed by washing with lysis buffer. Bound proteins were eluted by addition of Laemli buffer and were analyzed by SDS-PAGE followed by staining in Coomassie Blue or were subjected to western blot analysis employing the R2680 anti-dynamin 2 antibody (IGBMC; [42]).

## Supporting Information

Figure S1 **CNM mutations impact on dynamin 2′s localization to microtubules.** COS-1 cells transiently transfected with the indicated constructs were treated with Brinkley buffer and 1% (v/v) Triton X-100, followed by fixation in parafomaldehyde and staining with anti-dynamin 2 and anti-β tubulin specific antibodies. Dynamin 2 mutants (R522H, P627H) do not localize to microtubules but decorate punctate structures following MT enrichment.
(TIF)

Figure S2 **Effect of dynamin mutants**' **overexpression on transferrin uptake.** COS-1 cells expressing ectopic wild type and mutant dynamin 2 constructs were incubated with fluorescently labeled transferrin for 15 min and were subsequently fixed and processed with anti-MYC antibodies for microscopic observation. Representative images employed for quantification experiments described in Figure 4.
(TIF)

Figure S3 **Localization of dynamin 2 mutant constructs to clathrin-positive structures following a vesicular enrichment fixation.** COS-1 cells were transiently transfected with indicated dynamin 2 mutant constructs. Conditional dynamin knock-out cells were left untreated (CKO) or were treated with 4-hydroxytamoxifen for deletion of *Dnm1* and *Dnm2* (DKO). Cells were treated with 0.5% (v/v) Triton X-100, 2% (v/v) paraformaldehyde for 2 min at 37°C followed by fixation in paraformaldehyde and processing with anti-MYC specific antibodies and anti-clathrin light chain (CLC) antibodies. Samples were analyzed by confocal microscopy. A mask for the colocalization was created employing the Fiji software is shown (right panels). Note the presence of enlarged vesicles (arrows) in the case of R522H and P627H mutant expressing cells.
(TIF)

Figure S4 **CNM and CMT dynamin 2 mutations do not impact on BIN1 (amphiphysin 2) interaction.** Recruitment of dynamin 2 constructs to BIN1-induced membrane tubules. COS-1 cells were transiently transfected with vectors encoding indicated dynamin 2 constructs and the GFP-BIN1 chimera. Cells were fixed in paraformaldehyde followed by staining with anti-MYC specific antibodies. Both CNM and CMT dynamin 2 mutants can be seen in association with BIN1 tubules.
(TIF)
