# Supplementary figures and images for: Mild Functional Differences of Dynamin 2 Mutations Associated to Centronuclear Myopathy and Charcot-Marie-Tooth Peripheral Neuropathy
Source: PLoS One. 2011 Nov 11;6(11):e27498. doi: 10.1371/journal.pone.0027498 (PMC3214065; doi:10.1371/journal.pone.0027498)

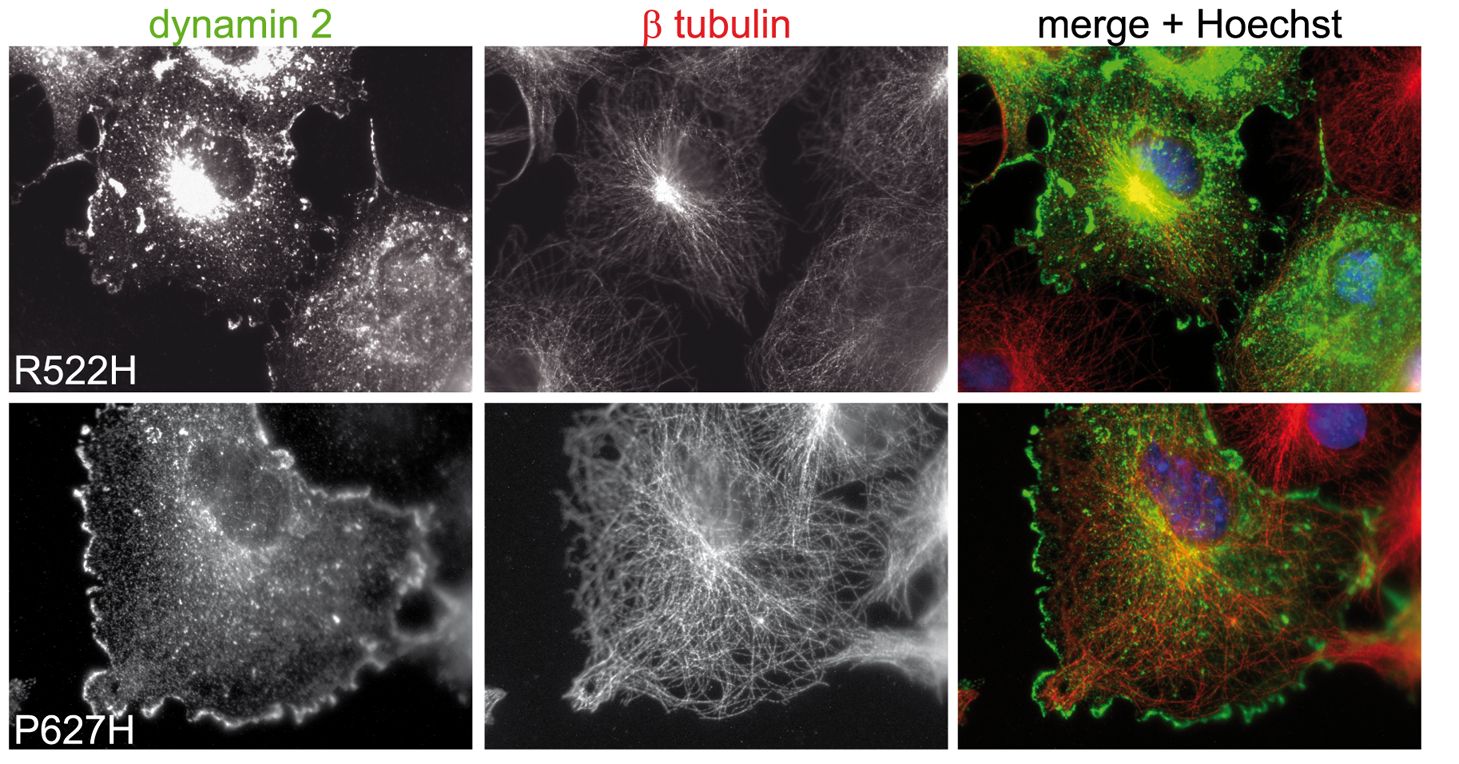

Supplement: Figure S1 — CNM mutations impact on dynamin 2′s localization to microtubules. COS-1 cells transiently transfected with the indicated constructs were treated with Brinkley buffer and 1% (v/v) Triton X-100, followed by fixation in parafomaldehyde and staining with anti-dynamin 2 and anti-β tubulin specific antibodies. Dynamin 2 mutants (R522H, P627H) do not localize to microtubules but decorate punctate structures following MT enrichment. (TIF) [file pone.0027498.s001.tif]

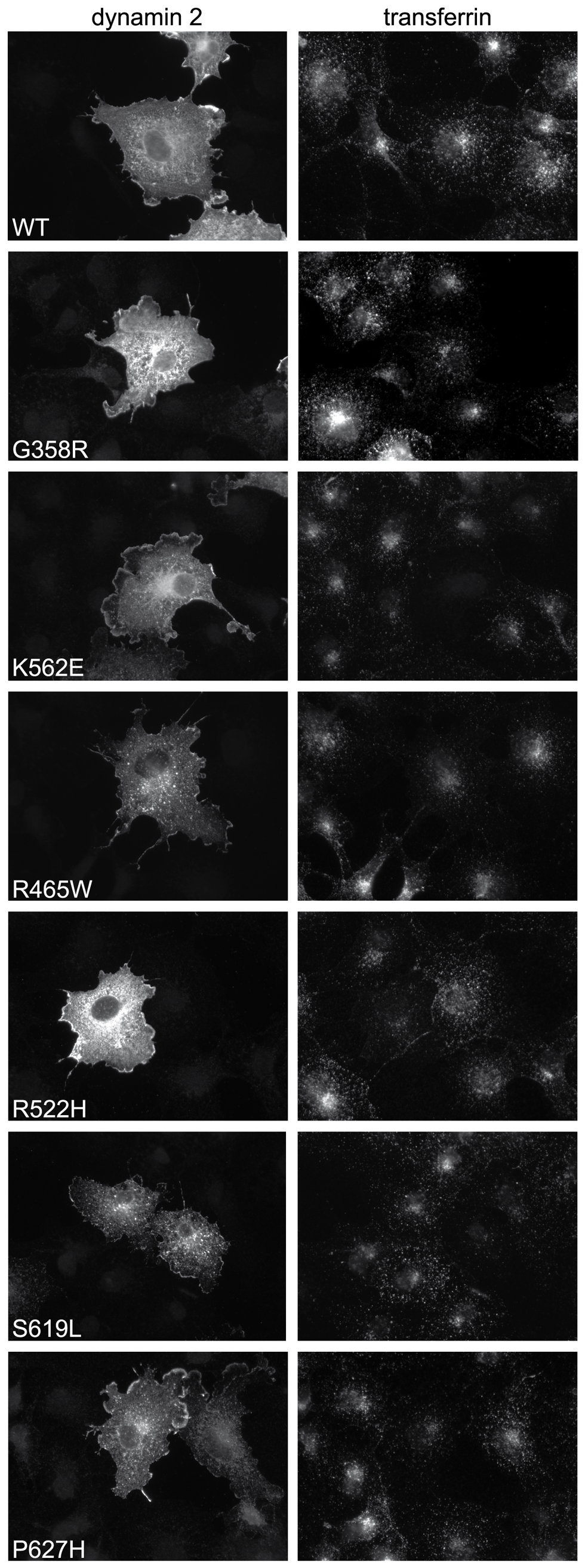

Supplement: Figure S2 — Effect of dynamin mutants' overexpression on transferrin uptake. COS-1 cells expressing ectopic wild type and mutant dynamin 2 constructs were incubated with fluorescently labeled transferrin for 15 min and were subsequently fixed and processed with anti-MYC antibodies for microscopic observation. Representative images employed for quantification experiments described in Figure 4. (TIF) [file pone.0027498.s002.tif]

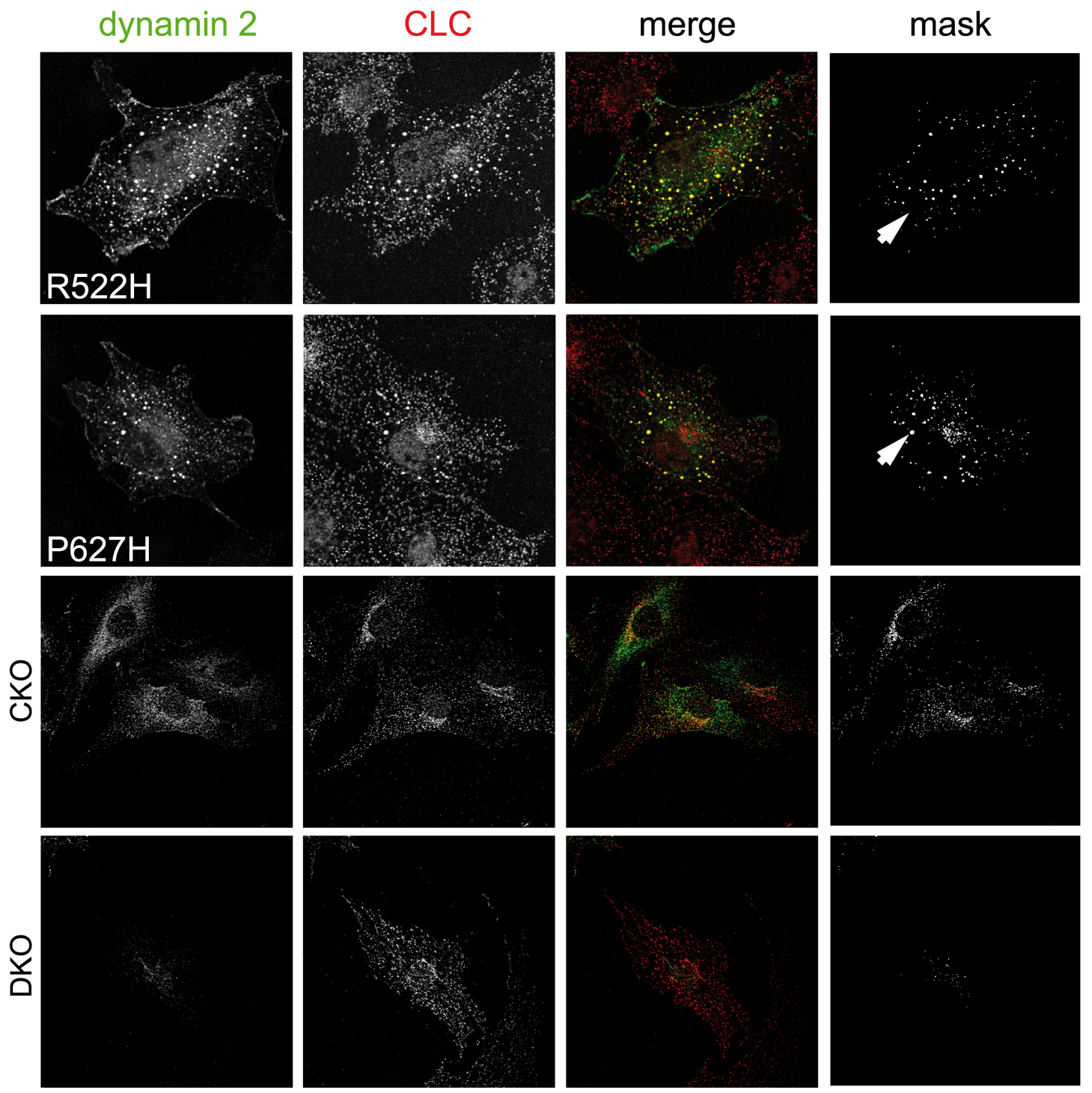

Supplement: Figure S3 — Localization of dynamin 2 mutant constructs to clathrin-positive structures following a vesicular enrichment fixation. COS-1 cells were transiently transfected with indicated dynamin 2 mutant constructs. Conditional dynamin knock-out cells were left untreated (CKO) or were treated with 4-hydroxytamoxifen for deletion of Dnm1 and Dnm2 (DKO). Cells were treated with 0.5% (v/v) Triton X-100, 2% (v/v) paraformaldehyde for 2 min at 37°C followed by fixation in paraformaldehyde and processing with anti-MYC specific antibodies and anti-clathrin light chain (CLC) antibodies. Samples were analyzed by confocal microscopy. A mask for the colocalization was created employing the Fiji software is shown (right panels). Note the presence of enlarged vesicles (arrows) in the case of R522H and P627H mutant expressing cells. (TIF) [file pone.0027498.s003.tif]

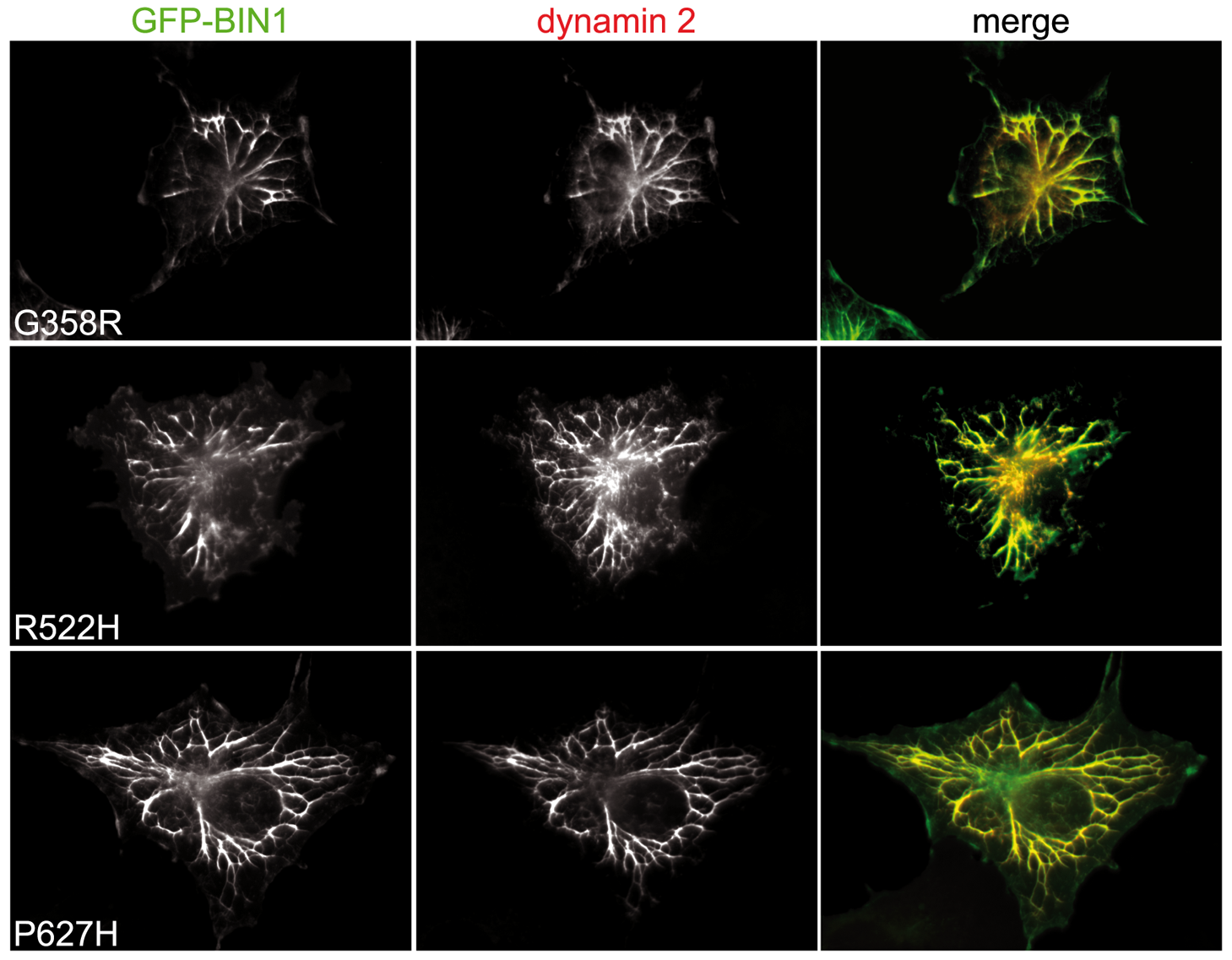

Supplement: Figure S4 — CNM and CMT dynamin 2 mutations do not impact on BIN1 (amphiphysin 2) interaction. Recruitment of dynamin 2 constructs to BIN1-induced membrane tubules. COS-1 cells were transiently transfected with vectors encoding indicated dynamin 2 constructs and the GFP-BIN1 chimera. Cells were fixed in paraformaldehyde followed by staining with anti-MYC specific antibodies. Both CNM and CMT dynamin 2 mutants can be seen in association with BIN1 tubules. (TIF) [file pone.0027498.s004.tif]
